# Collective behaviours in organoids

**DOI:** 10.1016/j.ceb.2021.06.006

**Published:** 2021-10

**Authors:** Qiutan Yang, Prisca Liberali

**Affiliations:** 1 Friedrich Miescher Institute for Biomedical Research (FMI), Maulbeerstrasse 66, 4058 Basel, Switzerland; 2 University of Basel. Petersplatz 1, 4001 Basel, Switzerland

**Keywords:** Collective behaviors, Organoids, Cell migration, Oscillation, Cell competition, Mechanics

## Abstract

Collective behaviour emerges from interacting units within communities, such as migrating herds, swimming fish schools, and cells within tissues. At the microscopic level, collective behaviours include collective cell migration in development and cancer invasion, rhythmic gene expression in pattern formation, cell competition in homeostasis and cancer, force generation and mechano-sensing in morphogenesis. Studying the initiation and the maintenance of collective cell behaviours is key to understand the principles of development, regeneration and disease. However, the manifold influences of contributing factors in *in vivo* environments challenge the dissection of causalities in animal models. As an alternative model that has emerged to overcome this difficulty, *in vitro* three-dimensional organoid cultures provide a reductionist approach yet retain similarities with the *in vivo* tissue in cellular composition and tissue organisation. Here, we focus on recent progresses in studying collective behaviours in different organoid systems and discuss their advantages and the possibility of improvement for future applications.

## Introduction

**Figure 1:**
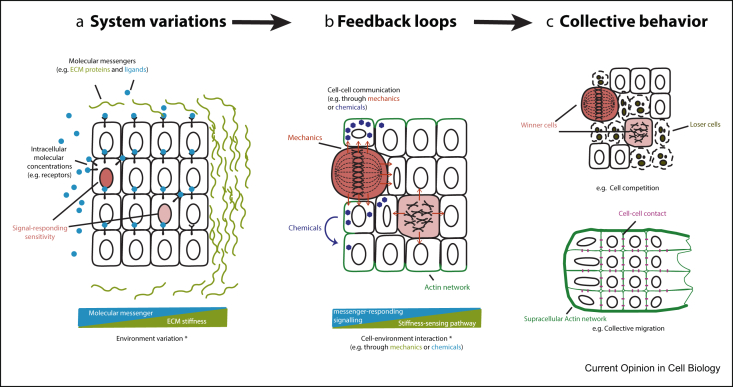
**The emergence of collective behaviour in the multicellular system**. **(a)** The initial variations of the multicellular system in the tissue context. Cells have different intracellular molecular concentrations (e.g. number of receptors) that determine the signal-responding sensitivity of each cell. In environment, the molecular messengers (e.g. ligands or extracellular matrix [ECM] protein) are unequally distributed. The mechanical cues from environment, such as the ECM stiffness, are variable as well. **(b)** Feedback loops that amplify initial system variations. Initial variations through the signalling cascade can induce different cellular behaviours, such as cells dividing at different time, secreting (or not secreting) different amount of molecules and establishing different protein expression patterns (e.g. actin pattern that associated with cell polarity). Cell–cell communication or cell–environment interaction happens through mechanical or chemical signals, which again triggers different signal-responding pathways and further diverses the cell groups. Feedback loops here exist at different scales, at molecular scale between signalling cascades, at cellular scale between different cells and at tissue scale between environment and the cell population. **(c)** Collective behaviours that through feedback loops raised from system variations. Two types of collective behaviours, the cell competition and the collective cell migration, are demonstrated as examples. ∗ in A and B indicates that the blue and green triangles above do not represent antagonistic relationship between molecular messenger and ECM stiffness, or between messenger-responding signalling and stiffness-sensing pathway, but illustrate the uneven distributions of biochemical and mechanical cues in the initial system variation **(a)** and the uneven activities of their responding pathways in feedback loops **(b)**.

Collective behaviours are emergent properties in complex systems that are composed of interconnected units. In multicellular systems, collective cell behaviours arise from the initial variable cell statuses that are nonlinearly amplified through feedback loops in cell–cell and cell–environment interactions. The initial variable cell statuses include fluctuations in intracellular molecular composition and microenvironments (Figure 1A), which through signalling cascade can induce more cellular differences, such as the different cell-cycle length, cell size, cell polarity, secretion and cell metabolism. As the building block of the multicellular system, cells interact with each other and the environment via chemical or mechanical signals (Figure 1B). The chemical-responding or mechano-sensing pathways generate more feedback to cell subpopulations, amplifying their differences and eventually leading to collective cell behaviours (Figure 1C) [1]. Because of the complexity on the multidimensional initial variations and feedback loops, quantifying features of the system at molecular, cellular and tissue scales is critical to illustrate the progress of collective cell behaviours (Table 1) [2]. Accordingly, model-based prediction, local perturbations and global perturbations on multiscale parameters are important to dissect the mechanism of collective cell behaviours (Table 1) [3, 4].

**Table 1:** Techniques suitable for studying collective behaviours.

Name	Purpose	Apply to which type of collective behaviour	Reference
1. Methods that measure or perturb systematically			

*In situ* RNA sequencing	Detect single-cell transcription profiling in preserved tissue.	General	[52]
Single-cell RNA sequencing	Map temporospatial single-cell transcription profiling in tissue.	General	[2, 53, 54]
Multiplex staining	Generate subcellular protein/RNA profiling.	General	[53, 55]
Image segmentation and feature extraction.	Generate the database for multivariate information including gene expression, mechanics and cell and tissue morphology.	General	[31, 53, 56]
Trajectory analysis	Building pseudo-time-lapse of tissue development.	General	[53]
Light-sheet microscopy	Long-term time-lapse recording of tissue dynamics.	General	[31, 53]
Theoretical modelling	Test dominant factors or parameters that regulate collective behaviours (e.g. vertex model, finite model, agent-based model).	General	[31, 47, 57, 58]
Microscopy-based measurement of mechanics	Generate force maps (e.g. Brillouin microscopy for viscoelastic properties, traction force microscopy for traction force on 2D culture).	Collective cell migration, force generation and mechano-sensing	[36, 59, 60, 61]
Synthetic ECM	Control mechanical and chemical cues in tissue environment.	Collective cell migration, force generation and mechano-sensing	[32, 62]
Microfluidic system	Mimic physiological rhythms and generate *in vivo*-like geometric confinement.	Oscillation, collective cell migration, force generation and mechano-sensing	[63]
Micropattern or bioengineered scaffold	Perturb or control of mechanical and chemical cues in tissue environment.	Force generation and mechano-sensing	[46, 48, 64, 65, 66]
2. Methods that measure or perturb locally			

Fluorescent indicator	Real-time visualise the dynamic process. Different indicators mark different objects (e.g. fluorescent protein-based signalling or cell fate or organelle reporters, calcium indicator, membrane tension indicator).	General	[67, 68, 69]
Optogenetic tool	Locally perturb gene expression or protein function and create different cellular behaviours in the subregion of tissue.	General	[70]
Microbead implantation	Induce local inhibition or activation of signalling with the attached molecular messenger (e.g. inhibitors or morphogens).	General	[71]
Laser cutting	Locally measure and perturb tissue or cell membrane tension.	Force generation and mechano-sensing	[31]
Micropipette aspiration	Measure cell membrane tension or tissue tension.	Force generation and mechano-sensing	[31, 72]
Lumen pressure measurement	Measure lumen hydraulic pressure.	Force generation and mechano-sensing	[72]
Microdroplet	Quantify anisotropic stress within tissue.	Force generation and mechano-sensing	[73]
Atomic force microscopy	Apply and measure forces on the defined region.	Force generation and mechano-sensing	[74]

In past decades, mechanisms on the emergence and maintenance of collective behaviours have been revealed in populations of unicellular organisms, such as bacterial and amoebae culture [5, 6]. In multicellular organisms, collective cell behaviours are the indispensable mechanism driving development and maintaining homeostasis, yet they are more complicated to dissect because of influences from the *in vivo* environment. To address the tissue-specific collective behaviours, isolated culture conditions, for instance *in vitro* or *ex vivo* tissue cultures, are necessary. Traditional two-dimensional (2D) cell culture experiments that are performed with homogenous populations of transformed cells can display a wide range of collective cell behaviours such as collective cell migration (CCM) and cell jamming [7, 8]. However, they lack heterogeneity in cell type composition that can drive more complex behaviours. On the other hand, some 2D tissue culture systems retain phenotypes that recapitulate *in vivo* behaviours (e.g. *ex vivo* mouse somite cultures that retain gene oscillation) and were used to address specific questions in collective behaviour [9]. Most of the collective behaviours emerge with proper three-dimensional (3D) tissue organisation and cellular composition, which can be provided by the 3D organoid cultures. In 3D organoid culture conditions, with tissue-specific morphogens and growth factors, stem cells (e.g. embryonic stem cells, induced pluripotent stem cells and tissue-specific adult stem cells) embedded in Matrigel or other conditions undergo tissue-specific differentiation and morphogenesis, evolving to organ-specific tissues. The organoids share similar cell composition, tissue morphology and tissue functionality with their *in vivo* counterparts (for reviews of advances in organoid systems [10, 11, 12, 13, 14, 15, 16]). Importantly, it has been shown that different collective cell behaviours were replicated in organoid cultures [17, 18, 19, 20,23, 24, 25,27,30,32]. In this review, we focus on the progress of using different organoid systems to study four types of collective cell behaviours: the CCM, the oscillation, the cell competition and the force generation and mechano-sensing.

## Collective cell migration

**Figure 2:**
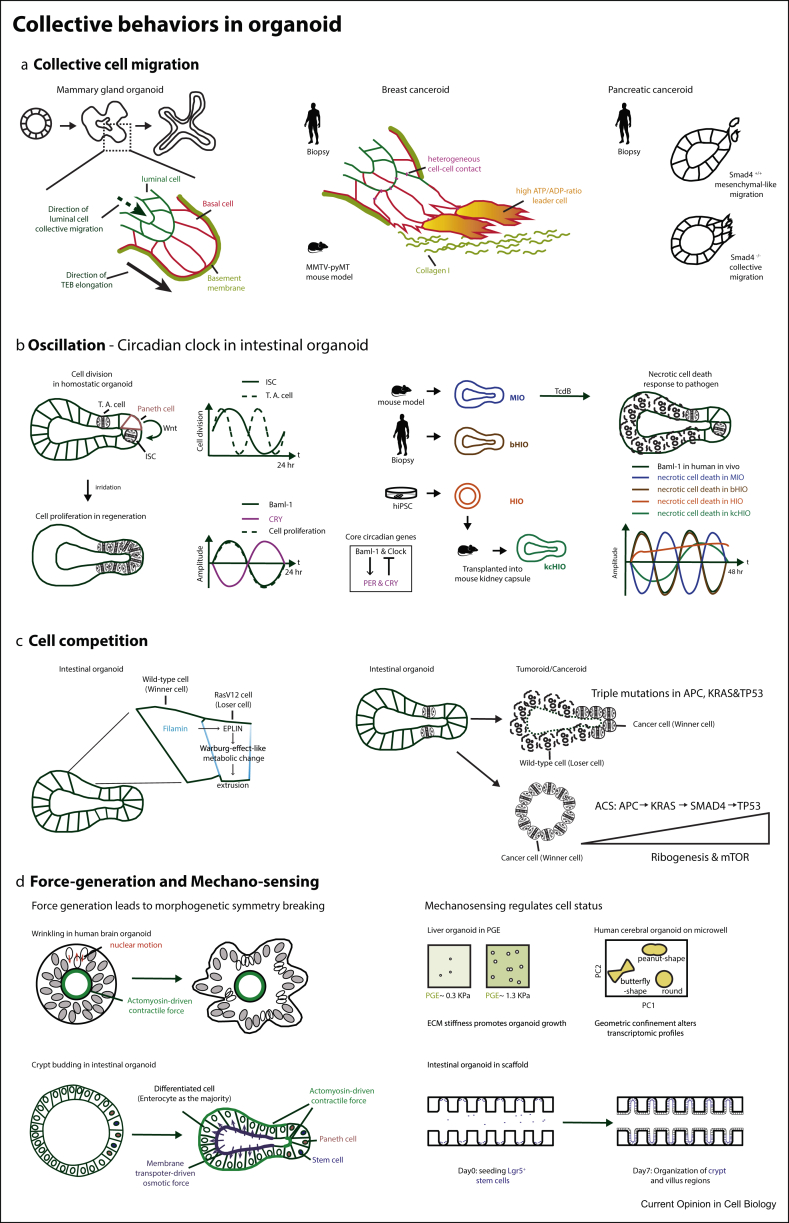
Collective behaviours in organoids. **(a)** Studies of collective cell migration in the mammary gland organoid, breast canceroid and pancreatic canceroid (see the main text, section of Collective Cell Migration for details). **(b)** Studies of the specific oscillation, the circadian clock, in the intestinal organoid (see the main text, section of Oscillation for details). ISC, intestinal stem cells; T. A. cell, transient amplifying cell; hiPSC, human induced pluripotent stem cell; MIO, mouse intestinal organoid; TcdB, *Clostridium difficile* toxin B; hr, hours. **(c)** Studies of cell competition in the intestinal organoid (see the main text, section of Cell Competition for details). EPLIN, epithelial protein lost in neoplasm; ACS, adenoma–carcinoma sequence. **(d)** Studies of force generation in the morphogenesis of the human brain organoid and mouse intestinal organoid and mechano-sensing events that regulate organoid growth in the mouse liver organoid, human cerebral organoid and mouse intestinal organoid (see the main text, section of Force generation and Mechano-sensing for details). ECM, extracellular matrix; PC, principal component; PEG, polyethylene glycol.

CCM (Box 1A, basics for CCM) is a typical collective behaviour that emerged in the multicellular environment and has been extensively studied in the fields of biophysics, developmental biology, regeneration and cancer research. Both the apical (e.g. regulation of cell–cell junctions) and basal (e.g. cryptic lamellipodia and integrin-based adhesions) dynamics in migrating cells have been revealed to drive collective migration, which attracts attention to the phenotypical analysis of CCM in time-lapse 3D at not only macroscopical but also single-cell resolutions [17, 33, 34, 35]. The 3D organoid culture is able to retain the microenvironments and morphology of tissues. Moreover, as the *in vitro* system, it is convenient to record and track organoid development at both the tissue level and single-cell resolution. Organoid cultures hence facilitate the study of CCM by providing image-based analysis at multiscale. A recent study combining intestinal organoid culture with 3D traction force measurement demonstrates rigorously that the gradients of cell apical and basal tensions along the axis from the stem cell niche to the differentiated region drive the CCM of intestinal epithelial cells [36]. Another CCM event is recapitulated in mammary gland organoids which display the transiently stratified terminal end bud (TEB) in the developing mammary tube. The TEB in mammary gland organoids is reminiscent to that observed in *in vivo* mammary placode. Interior cells of the TEB reduce epithelial polarity and cell adhesion and become protrusive and migratory that is similar to the phenotype of epithelial-to-mesenchymal transition [18]. A follow-up study using mammary gland organoid cultures indicates a cluster of cells at the TEB front with a high phosphorylated ERK1/2 level. These cells do not extend protrusions across basal cells to the Matrigel but migrate collectively towards the direction of tube elongation. Such CCM of the high-phosphorylated ERK1/2 cells is initiated and driven by Rac1 and MAPK/MEK signalling [19] (Figure 2A).

Box 1 **Basics for different collective behaviours**

A) Cell collective migration.

The prerequisites for CCM are the emergent signal of migrating directionality (e.g. in chemotaxis, haptotaxis, durotaxis and galvanotaxis) [57, 75], the intracellular responding machinery (e.g. responding through cytoskeleton arrangement and force generation) [75] and the intercellular coupling (e.g. via cell–cell contact, supracellular actomyosin cable, chemical/mechanical transmission through microenvironments) [33, 76]. The emergence of CCM has been addressed by *in silico* approaches that qualitatively mimic the critical parameters in CCM and combining with experiments that quantitatively validate and check the parameters [57, 58]. Through physical modelling, Camley and Rappel [57] have demonstrated interestingly that when tissue rheology (motion of individual cells) increases, the variation of cell responding capability/sensitivity within cell population can actually promote the accuracy of collective gradient sensing. A recent work of cellular-automaton–based modelling from Thuroff et al. [58] integrates multifeatures of single-cell migration into collective tissue dynamics, testing different CCM mechanisms and providing a generalised tool to stimulate different CCM scenarios.

B) Circadian clock

The circadian clock in mammals interacts with animal behaviours and regulates oscillatory physiological conditions such as metabolism and immune response. It leads to the 24-h day-to-night transcriptional rhythm of around 40% genes from genome. The suprachiasmatic nucleus of the hypothalamus receives light/dark input and generates physiological rhythms and therefore has been considered as the top of the circadian hierarchy. However, the peripheral circadian clocks, as a tissue-intrinsic property that emerges during cell differentiation and is independent of the suprachiasmatic nucleus, have also been detected in different tissues including heart muscles, the digestive track and its associated organs [25]. The core circadian clock genes are the E-box–binding transcriptional factors Baml-1 and Clock, which promote expression of PER and CRY that in turn inhibit Baml-1 and Clock [24].

C) Cell competition

By definition, cell competition must have at least two cells that differ genetically or have different concentration of gene products, which endows the winner cell advantage to actively eliminate the loser cell [77]. Natural difference in the growth rate of wild-type cell clones that allows two cell populations coexist and differ passively with time is not considered as cell competition [77]. The original discovery of cell competition is through artificial mutation of the Rp genes in the *Drosophila* imaginal disc [78]. Similar to Rp^+/+^ cells (winner) in Rp^+/-^ cell population (loser), gene mutations in different cases generate super-competitor cells that are essential to create competitive interaction despite the mechanisms of eliminating loser cells can be different [77]. In mammalian skin morphogenesis, the mechanism of eliminating loser cells changes from apoptosis to differentiation to adapt/facilitate the development of stratified tissue [79].

D) Force generation and Mechano-sensing

Physic features, especially mechanics, play important roles in coordinating cell–cell and cell–environment connections in collective cell behaviours, such as pushing and pulling forces that drive cell migration, tissue stiffness that guides migrating directionality, individual cell contractile forces that initiate tissue folding, sheering forces from blood or air flow that induce cell differentiation and branching morphogenesis and hydraulics that determine organ size and cell fates [3, 4].

Alt-text: Box 1

Besides being part of mammary gland development, CCM is a typical feature in breast cancer. Organoid cultures facilitate cancer research by using tumoroids/canceroids that are derived from biopsies or from tissues with oncogene mutations [37]. Mammary gland organoids generated from patient biopsies and the genetically engineered breast cancer MMTV-pyMT (the mouse mammary tumour virus long terminal repeat drives expression of the polyomavirus middle T oncogene) mouse model demonstrate that in collective invasion, the front leader cells lack common epithelial-to-mesenchymal transition markers and have heterogeneous cell–cell interaction with the follower cells. Collective cell invasion of breast luminal tumoroids requires the collagen-I-rich microenvironment and a switch of gene expression from the luminal cell type (interior cells in the TEB) to the basal cell type (exterior cells in the TEB) [20] (Figure 2A). In such a scenario, the leader–follower cell connection and the specific microenvironment are coordinated as a strategy for continued migration of cell groups. Revealed by organoid culture, another coordination in breast cancer CCM is regulated by metabolism and energy availability, in which the cells with higher glucose uptake and highest ATP/ADP ratio lead the CCM. The dynamic switch of leader positions facilitates cancer cells to overcome the energy-consuming environment during invasion [21] (Figure 2A). CCM as a typical type of cancer cell invasion has been also investigated in human pancreatic organoids. Interestingly, in pancreatic canceroids derived from 25 genetically different biopsies, SMAD4 mutation enables CCM through SMAD4-independent non-canonical TGF-beta signalling and its downstream targets of RAC1 and CDC42, whereas without SMAD4 mutation, the pancreatic cancer cells exhibit mesenchymal migration [22] (Figure 2A).

## Oscillation

Oscillations in multicellular systems emerge with at least two oscillators that feedback to each other and result in phasic dynamics of gene expression and related cellular activity in cell groups. Such examples exist in calcium pulsing–induced CCM during wound healing, NOTCH and FGF-guided somitogenesis during embryonic development and the development of the *in vitro* 3D-cultured gastruloid model [38, 39]. The most studied oscillation in organoids is the circadian clock in intestinal epithelium.

The circadian clock *in vivo* is set by anti-phasic accumulation and degradation of the core circadian proteins, Baml-1 and PER (Box 1B, basics for the circadian clock), in a rhythmic cycle of 24 h [40]. *In vitro* transformed mammalian cells such as Caco-2+ cells and NIH 3T3 cells display, on serum shock, lengthened oscillatory periods (>72 h) of Baml-1 expression with attenuated amplitude compared with *in vivo* tissues. While the transformed cell lines lack appropriate oscillations of the circadian clock, mature human and mouse intestinal organoids demonstrate a 24-h tissue-intrinsic circadian clock [23]. Three types of the circadian rhythm on collective cell behaviours have been found in intestinal organoids: (1) the timing of cell division in stem cells and progenitor cells in homeostatic organoids [24], (2) the cell proliferation in regenerative epithelium after irradiation [25] and (3) the necrotic cell death response to treatment of TcdB, a toxin produced by the intestinal pathogen *Clostridium difficile* [26]. In homeostatic organoids, the timing of cell division is coupled to the circadian clock through the rhythmic secretion of WNT in Paneth cells. Paneth cells therefore have been considered as the pacemakers for the oscillation of the circadian clock and cell division. Stem cells that sit next to Paneth cells have the 1:1 cell division–circadian clock coupling, whereas progenitor cells that are further away from the stem cell niche with diluted WNT concentration have 1:2 coupling (dividing faster) (Figure 2B) [24]. This study suggests that the coupling between the circadian clock and the cell cycle length of stem cells and progenitor cells leads to synchronised crypt formation in organoids [24]. Another example of circadian regulation happens during intestinal irradiation (e.g. in chemotherapy) that causes the loss of intestinal epithelium, which triggers tissue regeneration by cell proliferation of stem cells and progenitor cells. Such proliferation is coupled to the 24-h circadian clock through regulation of the core circadian clock gene Baml-1 on the production of cytokine TNF and chemokine CCL2, which further drive proliferation via the JNK pathway and regulate cell cycle timing via P21 [25] (Figure 2B).

Mouse intestinal organoids develop 24-h periodic gene expression of Bmal-1 and Per, whereas in human intestinal organoids, the core circadian gene expressions are determined by the maturity of tissue. The maturity of three types of human intestinal organoids, the human intestinal organoid derived from induced pluripotent stem cells (HIO), the HIO transplanted into the mouse kidney capsule (kcHIO) and the biopsy-derived human intestinal organoid (bHIO), is ranked as bHIO > kcHIO > HIO. On TcdB treatment, the coupling of the Baml-1 expression and circadian phasic necrotic cell death exists in bHIO and kcHIO but lacks in HIO, and the amplitude of Baml-1 expression in bHIO is higher than that in kcHIO. Interestingly, the peak of such necrotic cell death is 12 h different between human and mice organoids, which is possibly linked with the anti-phasic animal behaviours of diurnal humans and nocturnal mice. Combining organoid culture and RNAseq analysis, hundreds of genes have been indicated with circadian rhythmic expression, which is 10 times more than that identified from 2D cell cultures. Among these genes, Rac-1, as the direct target of TcbB, exhibited anti-phasic rhythmic expression between human and mice organoids [26].

## Cell competition

Cell competition is a type of collective cell behaviour that emerged in the multicellular system through winner–loser cell interaction (Box 1C, basics for cell competition). In intestinal organoids, through cell interaction, the oncogenic mutation RasV12 triggers the process of epithelial defence against cancer, in which the wild-type cells become winners and feedback to extrude the neoplastic RasV12 cells apically (Figure 2C) [27, 41]. Epithelial defence against cancer in wild-type winner cells accumulates filamin, which through competitive cell interaction recruits EPLIN (the actin-binding epithelial protein lost in neoplasm) in the RasV12 cells and triggers downstream Warburg effect–like metabolic change. The Warburg effect–like metabolic change upregulates glycolytic pathways and PDK-mediated mitochondrial dysfunction, eventually enhancing the elimination of the RasV12 cells (Figure 2C) [27]. During cancer progression, more or different oncogenic mutations make the cancer cells become winner cells. Combing intestinal organoid culture and RNAseq, mutations in the adenoma–carcinoma sequence of APC, KRAS, SMAD4 and TP53 have been shown to lead to a global transcriptional reprogramming towards ribogenesis and upregulated mTOR signalling, resulting in elevated cell proliferation [28]. In such adenoma–carcinoma progression, the winning strategy of cancer cells is to become hyper-proliferative and occupying the space and resources of wild-type cells (Figure 2C). Interestingly, another study with the same triple mutations on APC, KRAS and TP53 but lack of SMAD4 mutation revealed a different cancer cell competing strategy that is through inducing apoptosis in wild-type cells (Figure 2C) [29].

## Force generation and mechano-sensing

Force generation and mechano-sensing in cells create feedback between biophysical and biochemical signalling, establishing coordination between cellular mechanics and gene expression, a cell and its neighbouring cells and tissue and the extracellular environment in the system (Box 1D, basics of force generation and mechano-sensing). These feedback and connections in complex systems (e.g. the biological system of organoid cultures) lead to the emergence of collective behaviour such as self-organised morphogenesis (for review of mechanobiology in organoid [42, 43]).

A typical strategy studying the force-driving emergence of collective behaviour follows the steps of (1) analyse phenotype and select the critical parameters (e.g. biomechanics or biochemicals), (2) construct a theoretical model based on the measured parameters, stimulate and predict the phenotype and (3) experimentally validate model prediction and refine the theory [30, 31, 42, 44]. Without influence from other organs, organoid cultures provide a unique system, of which the initial status (e.g. cell numbers, energy status, medium concentration) can be defined. Thus, it simplifies the design of the theoretical model for the morphogenesis of studied organs by reducing the parameters. Combining experiments and physical modelling, the emergence of surface wrinkles in the human brain organoid, which is reminiscent of human brain wrinkling, has been analysed from the physical standpoint. The surface wrinkles are caused by mechanical instability as a consequence of tissue contraction in the centre region of the organoid and of the nuclear motion caused by residual stress on the peripheral region (Figure 2D) [30]. Another example is the study of mechanics-driven crypt morphogenesis in the intestinal organoid. In mouse intestinal organoid culture, crypt, the tissue compartment which contains stem cells, forms a budding structure that is similar to *in vivo* crypt morphology. Along with cell differentiation, distinct tissue mechanics are generated in crypt and villus regions [31, 36]. The crypt has relatively high apical tension and low basal tension, whereas the villus has high basal tension and low apical tension. Such different tension distributions are regulated by differential patterns of actomyosin in stem cells (from the crypt region) and enterocytes (from the villus region), which determine differential spontaneous curvature of the tissue that is a critical physical parameter driving crypt budding in the theoretical model. Moreover, during the generation of the tissue spontaneous curvature, the osmotic force in the system is also altered by enterocytes via membrane transporters. Specifically, sodium–glucose co-transporter 1 (SGLT-1), which is located at the cell apical side and essential for the physiological function of absorption in enterocytes, relocates liquid from lumen space into villus cells, decreasing the organoid monolayer tension and thus promoting budding. Here, different layers of coordination between the mechano- and osmotic forces, the individual cell fate and tissue mechanics, the morphogenesis and organ physiological function are exquisitely coupled, guaranteeing crypt budding at a correct spatiotemporal position (Figure 2D) [31].

Apart from force generation, another aspect of collective cell behaviour is the feedback of mechanics to biochemical signals and cell status. To address the mechanism, a defined or controllable mechanical environment in organoid culture is required. In most of the organoid cultures, the stiffness, water contents and biological composition of Matrigel are inside the range of an *in vivo* environment that cells and tissues naturally encounter and therefore allow cells freedom to grow in 3D, which is essential to force generation and correct mechano-sensing at the tissue level. However, Matrigel has the drawbacks of undefined biological composition, lot-to-lot variations and turbulence of mechanical features. Recent alternative approaches culture the intestinal organoid as a monolayer [36, 45], which allow organoid growth on collagen I by providing a coated 3D scaffold [46]. Through synthetical approaches, intestinal and liver organoids can grow in polyethylene glycol hydrogels with defined stiffness and a clear recipe of extracellular matrix protein mix. In liver organoid culture, non-physiological matrix stiffness (0.3 kPa) leads to failure of organoid growth, whereas physiological stiffness (1.3–1.7 kPa) allows organoid formation through the mechano-sensing signalling cascade of integrin—Src family of kinases (SFKs)—yes-associated protein 1 (YAP) (Figure 2D) [32]. Geometric confinement of tissue generates stress pattern and therefore is a useful tool to investigate mechano-sensing mechanisms. Microwell-confined human cerebral organoids in star, triangle, butterfly, peanut and round shapes display significant difference in transcriptomic profiles and neural lineage specification (Figure 2D) [47]. Finally, using scaffold mimicking gut tubular structures with crypt-like microcavities allows long-term growth of mouse intestinal organoids in correct crypt/villus spatial distribution. Rare cell types in Matrigel-embedded intestinal organoids, such as enteroendocrine cells, tuft cells and M-like cells, are enriched in scaffold cultures, indicating the mechano-feedback from geometric confinement to cell fate in intestinal epithelium (Figure 2D) [48].

## Conclusion and perspectives

Organoid cultures facilitate the study of collective behaviours. The abovementioned four types of collective cell behaviours share a common feature, namely, that they emerge with tissue-specific cell composition, which is an essential condition provided by organoids. Moreover, organoid cultures support high-resolution image-based analysis of the spatiotemporal dynamics of cell–cell interaction within the studied tissue. Large sample numbers that are easily obtained from organoid culture promote the quantification at the global/-omics level and the systematic analysis of critical steps that collectively alter tissue features. Because of the convenience of image-based and quantitative analysis in organoid systems, new molecular mechanisms, such as metabolic regulation, have been deciphered in cell competition and CCM. The easy accessibility of organoid culture also allows fine measurements and perturbations of mechanics from the subcellular to the tissue scales, advancing the study of tissue morphogenesis and mechano-chemical feedback in the inner organ that is difficult to access in animal models. In addition, human organoids bring forward a unique opportunity to investigate the collective cell behaviours directly in human tissue. For instance, pancreatic organoids derived from patient samples revealed clinic-relevant mutations that can lead to different types of cancer migration. Furthermore, the study of oscillation in intestinal organoids shows anti-phasic expression of human and mouse circadian core genes and therefore further enhances the point that certain biological processes need direct investigation in human tissue by generating human organoids.

Despite many advantages of using organoids, a missing part in many of the current systems is the cross-tissue cell–cell interaction, which in the *in vivo* environment can trigger and maintain the collective cell behaviours. For example, geometric confinement can alter cell status through mechano-chemical feedback. In embryonic gut tissue, such confinement initially is coming from mesenchymal condensation underneath the gut epithelium [49]. Recent work from Verhulsel et al. [46] establishes the co-culture of the intestinal organoid with fibroblasts in 3D collagen I scaffold, reproducing the microenvironment of gut stromal and enabling the study of epithelial cell/fibroblast interactions. For the future, co-culture of fibroblasts, immune cellsand/or neuronal cells with organoids in Matrigel or engineered scaffolds, which is under constant development in the organoid field, will enrich the study of collective cell behaviour in a more tissue authentic context. Assembloids and organoid-on-chips will also bring new platforms to address the collective cell behaviours in the scenarios of inter-organ communication and host–pathogen interaction [14, 50, 51].

## Conflict of interest statement

No conflict of interest.
